# The influence of CePO_4_ nanorods on the CO oxidation activity of Au/GdPO_4_-rods†

**DOI:** 10.1039/c8ra02206b

**Published:** 2018-06-13

**Authors:** Yu Huanhuan, Chen Fayun, Zhubaolin, Huang Weiping, Zhang Shoumin

**Affiliations:** College of Chemistry and Environmental Science, Shangrao Normal University Shangrao 334001 P. R. China yuhuanhuan08@163.com +8615620206206; Department of Chemistry, Key Laboratory of Advanced Energy Material Chemistry (MOE), TKL of Metal and Molecule Based Material Chemistry, Nankai University Tianjin 300071 P. R. China zhangsm@nankai.edu.cn +8613920779712

## Abstract

In this work, Au/GdPO_4_-rods were found to be good catalysts for CO oxidation with a low content of Au. The dopant of CePO_4_ could influence the activity of Au/GdPO_4_ due to the synergistic effect. GdPO_4_ and CePO_4_ nanorods were obtained by a hydrothermal process and the Au/GdPO_4_-rod and Au/Ce-GdPO_4_-rod catalysts were prepared by deposition–precipitation synthesis. The samples were extensively characterized by transmission electron microscopy (TEM), inductively coupled plasma (ICP), powder X-ray diffraction (XRD), X-ray photoelectron spectroscopy (XPS), ultraviolet-visible spectroscopy (UV-Vis), Fourier transform infrared spectroscopy (FT-IR), temperature programmed desorption (O_2_-TPD, CO-TPD, and CO_2_-TPD) and N_2_ adsorption–desorption. The results showed that Au/GdPO_4_ with a low Au content possessed good activity for CO oxidation. When the content of Ce is 25 at%, 0.5% Au/Ce-GdPO_4_-rods can convert CO completely at 65 °C, and the catalyst showed better high-temperature resistance than 0.5% Au/GdPO_4_-rods. 0.5% Au/Ce-GdPO_4_-rods also showed good stability at reaction temperatures of 55 and 65 °C with CO conversions of 90% and 100% after continuous operation for 12 h. They also showed no deactivation after 50 h at a relative high reaction temperature of 200 °C.

## Abstract 2

A CePO_4_–GdPO_4_ composite was prepared by a general ultrasound method and could be a good support for gold nanocatalysts. Au/CePO_4_–GdPO_4_ catalysts with a low content of gold showed good catalytic activity, high temperature resistance and stability for CO oxidation.

## Graphical abstract

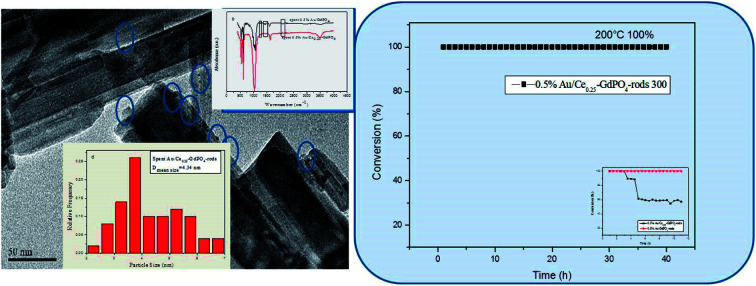

## Introduction

CO oxidation is one of the most extensively investigated reactions in heterogeneous catalysis due to its importance in both practical applications and fundamental studies, such as in CO removal from exhaust gas, environmental protection and air cleaning, and as probe reaction for the demonstration of catalytic mechanisms.^1–13^ It is of great importance to convert CO into non-toxic CO_2_ due to the CO poisoning effect. Catalytic CO oxidation is still one of the most effective methods for the elimination of CO.^6^ Common CO oxidation catalysts mainly involve metal oxide, noble metal, and alloy catalysts.^1,5,10^ Supported gold nanoparticles have been well studied for CO oxidation as one of the most active catalysts due to their excellent catalytic performance.^14–19^

For a long time, Au has been considered chemically inert and inactive in catalysis. However, since Haruta demonstrated that when Au was supported on a certain metal oxides, it showed high CO oxidation catalytic activity, Au nanocatalysts have triggered a great deal of research activities in the past decades.^18–26^ Many materials such as metal oxides and metal salts have been used as supports for noble metal catalysts, typically for Au catalysts.^14,19,27^ Most of them could not stand high temperature and have low stability. As known, the catalysts sometimes should be used at high temperature, so the catalyst must have high temperature resistance and stability. The deactivation and low stability have been the biggest drawbacks for Au catalysts' practical applications.^28,29^ How to develop feasible catalysts with excellent catalytic activity and stability for CO oxidation is still waiting to be solved. The major reason of the deactivation is often attributed to the agglomeration of Au nanoparticles, formation of carbonates adsorbed on the active sites, and change in oxidation state of Au species.^26,29^ References suggested that oxides or binary mixed oxides such as rare earth-TiO_2_,^30^ could help stabilize Au nanoparticles, which could prevent Au nanoparticles from sintering after deposition on the supports.^26–33^ More importantly in the composites, the advantages of the different phases could be combined to obtain strong surface interactions.^3,7,11,14,34,35^ It has been well established that the composition and structure of catalyst support play an important role in the catalytic performance. Tang *et al.* also found that hydroxyapatite/titanium-dioxide could stabilize gold nanoparticles due to the strong metal-support interaction.^33^ It significantly lowers the barrier to practical applications of supported Au catalysts, especially for high-temperature catalytic reaction.

Recently our group also found that gold nanoparticles supported on BiPO_4_ nanorods, LnPO_4_ (Ln = La, Ce) and nanosized YPO_4_ with low content of Au were extremely active for CO oxidation.^36–38^ However, the Au/YPO_4_-rods suffered severe deactivation after high temperature pretreatment. Therefore, we designed to use mixed CePO_4_–GdPO_4_ composite as supports *via* the synergistic effect between CePO_4_ and GdPO_4_ to obtain a type of high stable and sintering resistant Au supported catalyst.

In this work, CePO_4_ and GdPO_4_ nanorods were prepared by hydrothermal process. CePO_4_–GdPO_4_ composite was prepared by a general ultrasound method. By a deposition–precipitation process, gold catalysts were successfully prepared. The techniques of inductively coupled plasma (ICP), N_2_ adsorption–desorption, powder X-ray diffraction (XRD), transmission electron microscope (TEM), X-ray photoelectron spectroscopy (XPS), temperature programmed desorption (O_2_-TPD, CO-TPD, and CO_2_-TPD) and ultraviolet-visible spectroscopy (UV-Vis), Fourier transform infrared spectroscopy (FT-IR) were used for catalyst characterization. CO oxidation was selected as probe reaction to discuss the catalytic activity. The addition of CePO_4_ has a positive effect on the stabilization of gold particles, which helped the catalysts exhibit high catalytic activity, sintering resistance, and high stability.

## Experimental

All chemicals in this paper were of analytical grade, and they were used directly without any further purification.

### Support preparation

The CePO_4_ nanorods were synthesized by a hydrothermal method used in ref. 37, 39 and 40. In a typical synthesis, solutions of NH_4_H_2_PO_4_ (30 ml) and Ce(NO_3_)_3_ (30 ml) with molar concentration of 0.3 mol L^−1^ were mixed by vigorous stirring. 25 wt% ammonia was used to adjust the pH of the mixture to ∼2. Then the suspension was poured into a Teflon-lined stainless steel autoclave and kept at 160 °C for 14 h. After air-cooled to room temperature, the products were filtered, washed with deionized water and absolute alcohol. Finally, the white precipitate was dried at 80 °C overnight, then calcined at 400 °C for 3 h in air to get CePO_4_ nanorods.

The GdPO_4_ nanorods were synthesized by co-precipitation process. The calculated amounts of Gd(NO_3_)_3_·6H_2_O were mixed with 100 ml water–ethylene glycol solution (*V*_H_2_O_ : *V*_EG_ = 40 : 60). The obtained mixture was vigorously stirring for 1.5 h at 80 °C. Then 10 ml solution of NH_4_H_2_PO_4_ (0.05 g ml^−1^) was added to the above mixture. After that the suspension was refluxed at 140 °C for 3 h. Then the precipitate was washed with water and ethanol several times by centrifugation, dried at 80 °C overnight. The final white precipitate was calcined at 500 °C for 4 h in air with a heating rate of 5 °C min^−1^ to obtain GdPO_4_ nanorods.

CePO_4_–GdPO_4_ composites (Ce and Gd in the molar ratio of 5 : 95, 25 : 75, and 50 : 50) were obtained by an ultrasonic process. CePO_4_ and GdPO_4_ were mixed by milling 30 minutes. Then the mixture was dispersed in 100 ml H_2_O. After stirring 1 h, the suspension was then treated by ultrasonic dispersion for 2 h. Finally, the precipitates were centrifugated and dried at 80 °C overnight. The products were denoted as Ce_0.05_-GdPO_4_, Ce_0.25_-GdPO_4_, Ce_0.50_-GdPO_4_ with the concentration (in at%) of Ce as 5 at%, 25 at% and 50 at% respectively.

### Catalyst preparation

In the preparation procedure of all Au catalysts, Au was loaded by deposition–precipitation method. Firstly, 0.4 g supports were dispersed in 150 ml deionized water with stirring. After 2 h, amount of HAuCl_4_ (0.01 mol L^−1^) was added with the nominal content of Au as 0.1%, 0.3% or 0.5%. Then *via* an ultrasonic process, the supports were distributed well in the solution of HAuCl_4_. Afterwards, certain amount of CO(NH_2_)_2_ was added to adjust pH value of the above suspension to ∼9. Then the suspension was heated in water bath at 90 °C for 4 h. At last, the precipitate was centrifuged, and washed with deionized water several times. Finally, the obtained Au catalysts were calcined at 300 or 500 °C for 2 h in air with a heating rate of 5 °C min^−1^. The concentrations of Au were expressed as percentage by weight percent.

### Characterization techniques

The powder X-ray diffraction (XRD) study was carried out on a Rigaku D/Max-2500 X-ray diffractometer (kα *λ* = 0.154 nm) in the 2*θ* range of 3–80° to check the crystallographic phase purity of the samples. Transmission electron microscope (TEM) observations were obtained with a JEM-2100 or JEM-2010FEF transmission electron microscopes operating at 200 kV. X-ray photoelectron spectroscopy (XPS) data were recorded on a Kratos Axis Ultra DLD X-ray photoelectron spectrometer using a monochromated Al Kα source operated at 150 W to identify the chemical composition and the oxidation state of the catalysts. Gold loadings of the catalysts were determined by inductively coupled plasma-atomtic emission spectroscopy (IRIS Intrepid II XSP). Ultraviolet-visible spectroscopy (UV-Vis) spectra were recorded on a UV-3600 UV-Vis NIR spectrophotometer. Fourier transform infrared spectroscopy (FT-IR) spectra of the samples were obtained with a FTS 6000 spectrophotometer. Temperature-programmed desorption (TPD) data were recorded in a Quantachrome ChenBet TPR/TPD. 100 mg of sample was heated to 300 °C in He for 1 h. After cooling to room temperature, the samples were exposed to corresponding gas (CO, O_2_ or CO_2_) for adsorption until saturated. The relative desorption (CO, O_2_ or CO_2_) was performed in 15 ml min^−1^ He with a heating rate of 10 °C min^−1^ from 50 to 800 °C. A cold trap prior to the thermal conductivity detector (TCD) was used during the desorption processes. The desorbed CO, O_2_ or CO_2_ was measured by thermal conductivity detector.

The Brunauer–Emmett–Teller (BET) surface areas of samples were measured by N_2_ adsorption at liquid N_2_ temperature (77 K) using a Micromeritics apparatus. The samples were degassed at 473 K for 3 h prior to the adsorption experiments. The pore size was obtained from the adsorption branch of N_2_ isotherm by the BJH model.

### Catalytic activity tests

Catalytic activity evaluation was performed in a fixed-bed flow millireactor with an inner diameter of 8 mm. 200 mg of catalyst were diluted with 17.6 g chemically inert quartz sand. Subsequently, a mixture, 10% CO balanced with air was introduced into the reactor at a total flow rate of 36.3 mL min^−1^. After holding at the reaction temperature for 30 min, the gaseous products were onlined analyzed by CO_*x*_ analyzer (GC-508A gas chromatography). This evaluation method of catalytic activity was similar to that described by our group elsewhere.^36–38^

## Results and discussion

### ICP

Table 1 shows the measured Au loadings for Au/GdPO_4_-rods, Au/Ce-GdPO_4_-rods, and Au/CePO_4_-rods catalysts. The results suggested that Au loading efficiency of all catalysts was >70%. It almost reached 98% for 0.5% Au/CePO_4_-rods. After the addition of CePO_4_, Au loading efficiency increased. It revealed that Au species were more likely deposited on the surface of Ce-GdPO_4_ and CePO_4_. The synergistic effect could exist in Au/Ce-GdPO_4_. It is known that the Au-support interface largely depending on the deposition of Au particles is supposed to be an important factor for catalytic activity.^4*a*,35^ Thus, the deposition of Au can suggest the interaction between support and Au species. Here, from the results, it could be concluded that CePO_4_ could help enhance electrostatic attraction leading to high gold deposition. The results revealed that the metal support interaction was strengthened by doping CePO_4_.

**Table 1:** Actual Au contents in Au/GdPO_4_-rods and Au/Ce-GdPO_4_-rods catalysts

Samples	Actual Au loading (wt%)
0.1% Au/GdPO_4_-rods	0.07
0.3% Au/GdPO_4_-rods	0.21
0.5% Au/GdPO_4_-rods	0.41
0.5% Au/Ce_0.05_-GdPO_4_-rods	0.42
0.5% Au/Ce_0.25_-GdPO_4_-rods	0.45
0.5% Au/Ce_0.50_-GdPO_4_-rods	0.45
0.5% Au/CePO_4_-rods^37^	0.49

### XRD

**Fig. 1:**
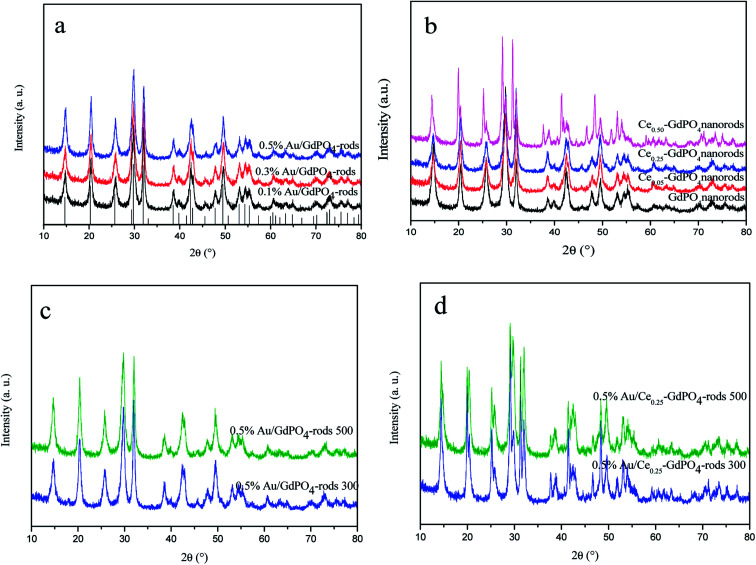
XRD patterns of Au/GdPO_4_-rods with different Au contents (0.1%, 0.3%, 0.5%) calcined at 300 °C for 2 h (a), GdPO_4_ and Ce-GdPO_4_ supports (b), 0.5% Au/GdPO_4_-rods and 0.5% Au/Ce_0.25_-GdPO_4_-rods calcined at 500 °C for 2 h (c) and (d).

XRD patterns of the samples are shown in Fig. 1. In Fig. 1a and b, for GdPO_4_ and Au/GdPO_4_ (with Au content: 0.1%, 0.3%, 0.5%) calcined at 300 °C, the main diffraction peaks observed at 14.8°, 20.4°, 25.8°, 29.8°, 32.0°, 38.6°, 39.8°, 42.4°, 42.8°, 47.8°, 49.5°, 53.0°, 54.3°, and 55.3°could be attributed to (100), (101), (110), (200), (102), (112), (210), (211), (003), (301), (212), (203), (302), and (310) planes of hexagonal GdPO_4_ (JCPDS 39-0232). If the Au content was too low, it is not very accurate to analyze the samples by XRD. After deposition of Au, for comparison, Fig. 1a showed the XRD pattern of the as-synthesized Au/GdPO_4_-rods. As predicted, there was no peaks belonged to metallic Au (2*θ* = 38.2 and 44.5°).^17*b*,19,29^ This could be due to low content and/or small particle size of gold nanoparticles which were lower than the detection limit of XRD. Thus, the Au size estimation could be obtained from TEM data. The X-ray powder diffraction patterns of CePO_4_–GdPO_4_ and GdPO_4_ supports are shown in Fig. 1b. With increasing the amount of CePO_4_, the reflections at 2*θ* = 25.2°, 29.2°, 38.9°, 46.7°, and 52.0°, were clearly assigned to hexagonal phases of CePO_4_. After the catalysts calcined at 500 °C (Fig. 1c and d), the intensity of the reflections slightly increased compared with the samples calcined at 300 °C, indicating the degree of crystallinity of the samples. Due to the high temperature treatment, Au particles may agglomerate to big particles which would have inhibited effect on the catalytic activity. However, no reflections assignable to the presence of Au were observed, indicating low content of Au species in the catalysts should have high dispersion.

### BET

**Fig. 2:**
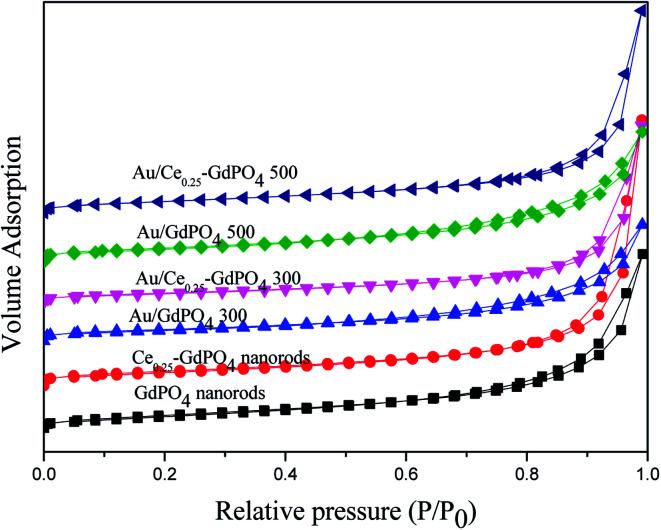
Nitrogen adsorption–desorption isotherms of GdPO_4_ nanorods, Ce_0.25_-GdPO_4_ composites and the supported Au catalysts.

The surface area and porosity of GdPO_4_ nanorods, Ce-GdPO_4_ nanorods (Ce: 5, 25, 50 at%), and the supported gold catalysts calcined at 300 °C and 500 °C for 2 h were characterized by N_2_ adsorption–desorption technique. As shown in Fig. 2, all the samples showed a distinct type IV nitrogen adsorption–desorption isotherm with H3-type hysteresis loops which were typical of mesoporous materials.^4*b*,6,7,13,28^ It could be seen in Table 2, after the deposition of gold, the BET surface areas and pore size of GdPO_4_ nanorods and Ce-GdPO_4_ nanorods changed, suggesting the block dispersed Au nanoparticles into the pores of the supports. With increasing the calcination temperature, the surface area of the catalysts decreased which may be due to the agglomeration of Au nanoparticles after high calcination temperature pretreatment. While Au/Ce-GdPO_4_-rods exhibited larger surface area than Au/GdPO_4_-rods, indicating that Au/Ce-GdPO_4_-rods was more sintering-resistant with smaller Au nanoparticles than Au/GdPO_4_-rods after calcination at 500 °C. This result could be also proved by the TEM data. Yet, the high surface area and pore volume were beneficial for providing more active sites enhancing catalytic activity the catalysts.^22,28,31^ As shown in Fig. S1,† CePO_4_ nanorods possessed the BET surface area of 27 m^2^ g^−1^. It is noteworthy that Ce-GdPO_4_ nanorods obtained higher surface area than pure GdPO_4_ and GdPO_4_. The above results showed that synergistic effect might exist in Ce-GdPO_4_ nanorods. It could be also concluded that due to the synergistic effect of CePO_4_ between gold nanoparticles and the support in Au/Ce-GdPO_4_-rods catalysts, Au/Ce-GdPO_4_-rods calcined at elevated high temperature showed larger surface area than Au/GdPO_4_-rods, which would help enhance the activity. Combined with ICP data, we could conclude that this strong interaction in Au/Ce-GdPO_4_ would increase adsorption capacity of Ce-GdPO_4_ for Au species. Thus, Au loading efficiency was increased and higher than Au/GdPO_4_ enhancing the activity.

**Table 2:** BET specific surface characteristics of the supports and catalysts

Samples	BET surface area (m^2^ g^−2^)	Average pore size (nm)	Pore volume (cm^3^ g^−2^)
GdPO_4_ nanorods	64.87	7.98	0.24
Ce_0.25_- GdPO_4_ nanorods	70.43	6.91	0.36
0.5% Au/GdPO_4_ 300	58.90	6.84	0.18
0.5% Au/Ce_0.25_-GdPO_4_ 300	58.45	7.22	0.26
0.5% Au/GdPO_4_ 500	40.58	7.88	0.21
0.5% Au/Ce_0.25_-GdPO_4_ 500	50.67	7.33	0.28

### TEM

**Fig. 3:**
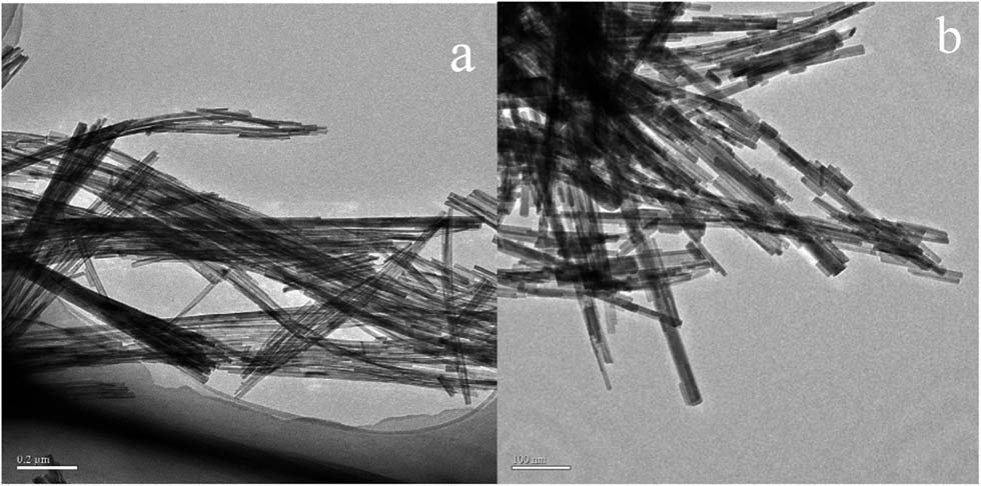
TEM images of GdPO_4_-rods (a) and Ce_0.25_-GdPO_4_-rods (b).

**Fig. 4:**
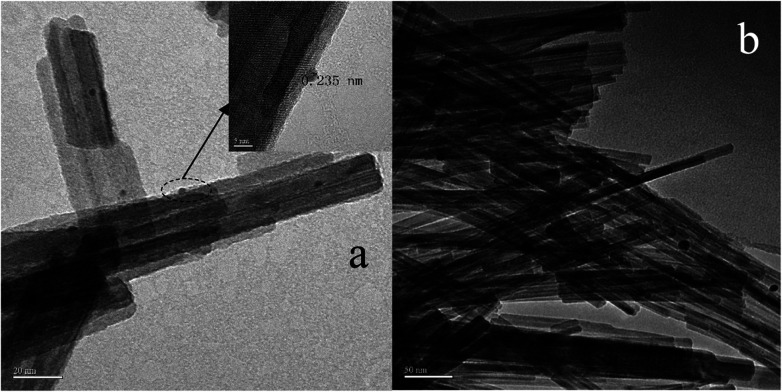
TEM images of fresh 0.5% Au/GdPO_4_-rods (a) and spent 0.5% Au/GdPO_4_-rods calcined at 300 for 2 h.

**Fig. 5:**
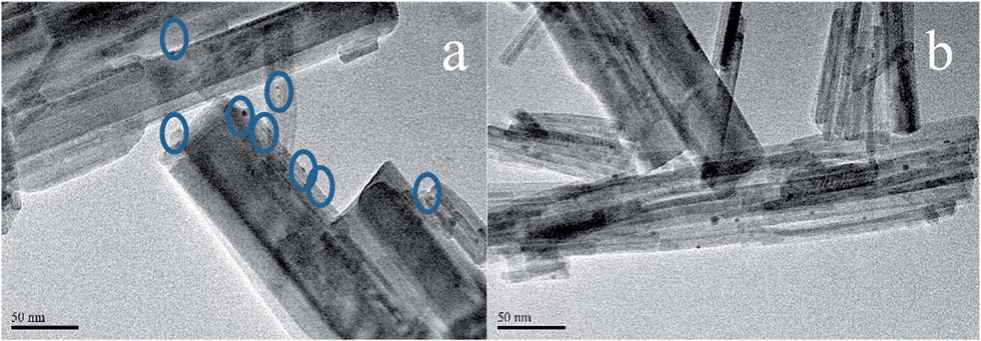
TEM images of fresh 0.5% Au/Ce_0.25_-GdPO_4_-rods (a) and spent 0.5% Au/Ce_0.25_-GdPO_4_-rods (b) calcined at 300 °C for 2 h.

**Fig. 6:**
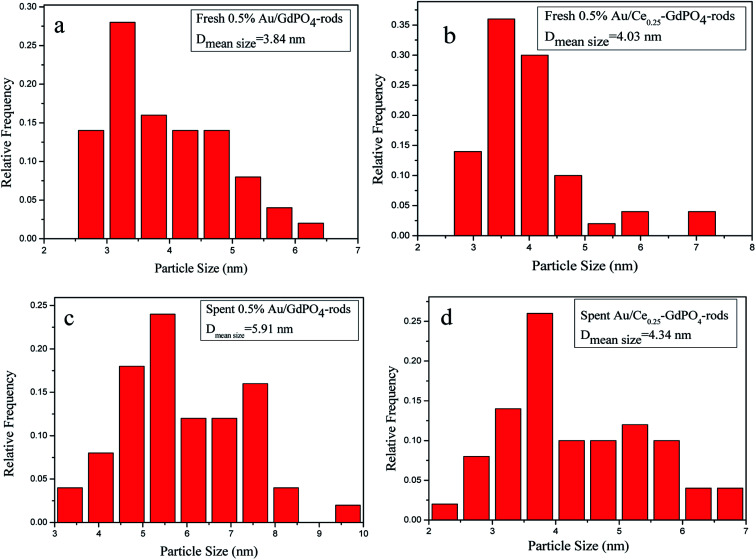
Size distribution of catalysts (a) fresh 0.5% Au/GdPO_4_-rods, (b) fresh 0.5% Au/Ce_0.25_-GdPO_4_-rods, (c) spent 0.5% Au/GdPO_4_-rods, and (d) spent 0.5% Au/Ce_0.25_-GdPO_4_-rods.

**Fig. 7:**
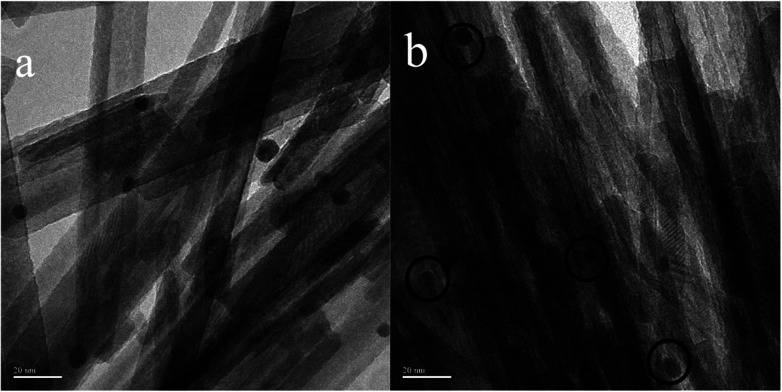
TEM images of 0.5% Au/GdPO_4_-rods (a) and 0.5% Au/Ce-GdPO_4_-rods (b) calcined at 500 °C for 2 h.

In order to determine the size distribution of Au nanoparticles, TEM and high-resolution (HR)-TEM (HRTEM) images were carried out. In Fig. 3a, GdPO_4_ presents the morphology of rods with the width of 0.5–1.0 μm and the diameter of 8–15 nm. After the addition of CePO_4_, a homogeneous composite of CePO_4_ and GdPO_4_ nanorods was obtained (Fig. 3b). The lattice spacings (inset of Fig. S2a and b†) of CePO_4_ nanorods and GdPO_4_ nanorods were observed from the HRTEM images. As shown in Fig. S2c,† after CePO_4_ was added in GdPO_4_, it was really hard to distinguish which rod was GdPO_4_ from TEM image, because of their similar morphology. In the HRTEM image (inset of Fig. S2c†), the crystalline plane spacings of CePO_4_ and GdPO_4_ respectively decreased a little in comparison with pure CePO_4_ and GdPO_4_. Combining with EDX results of Fig. S3,† it could be confirmed that CePO_4_ nanorods had dispersed well among GdPO_4_ nanorods and strongly interacted with GdPO_4_ nanorods. Fig. 4 shows the TEM results of fresh and Au/GdPO_4_-rods calcined at 300 °C for 2 h. Fig. 5 shows the TEM pictures of fresh and spent Au/Ce_0.25_-GdPO_4_-rods. It could be found that the gold nanoparticles which could be determined by the crystalline plane spacing of 0.235 nm (inset of Fig. 4a) assigned to the (111) plane of Au species^32^ with the mean size of about 3.84 nm (Fig. 6a) highly dispersed on the surface of GdPO_4_ nanorods. The Au nanoparticles with the average diameter of about 4.03 nm (Fig. 6b), which was slightly bigger than that of Au/GdPO_4_-rods, also dispersed well on the surface of Ce_0.25_-GdPO_4_ (Fig. 5a). However, due to the existence of synergistic effect, mean particle size of Au nanoparticles in Au/Ce-GdPO_4_-rods was a bit bigger than Au/GdPO_4_-rods. ICP data suggested that there were more Au species deposited on the surface of Ce-GdPO_4_ nanorods than GdPO_4_ nanorods. Thus, Au species in Au/Ce-GdPO_4_-rods might aggregate more easily than that in Au/GdPO_4_-rods, subsequently resulting in the large particle size of Au nanoparticles. In Fig. 4b and 5b, for the spent catalysts, it's clear that Au nanoparticles in spent Au/GdPO_4_-rods agglomerated leading to the growth of some big particles with the mean size of 5.91 nm (Fig. 6c), which would decrease the activity of Au/GdPO_4_-rods. And in the spent Au/Ce_0.25_-GdPO_4_-rods, few Au particles sintered during the reaction of catalytic CO oxidation as expected (Fig. 6d). After the catalysts were calcined at 500 °C, the Au nanoparticles in Au/GdPO_4_ sintered severely as the size of Au nanoparticles dramatically increasing to 8.0 nm (Fig. 7a). However, for Au/Ce_0.25_-GdPO_4_-rods, the size of Au nanoparticles increased to only about 5.0 nm (Fig. 7b). The explanation for the TEM results could be that the synergistic effect between CePO_4_ and GdPO_4_, which could help stabilize Au nanoparticles, so after continuation running in catalytic CO oxidation reaction and high temperature treatment, gold particles in the catalysts could still exist as small particles. As known, Au nanoparticles with the diameter of <5 nm could have good activity.^11,22,30^ Indeed, Au/Ce-GdPO_4_-rods calcined at 500 °C exhibited better activity than Au/GdPO_4_. The ICP and BET results also proved that synergistic interaction of CePO_4_ had a positive effect on the stability of Au nanoparticles.

### O_2_-TPD

**Fig. 8:**
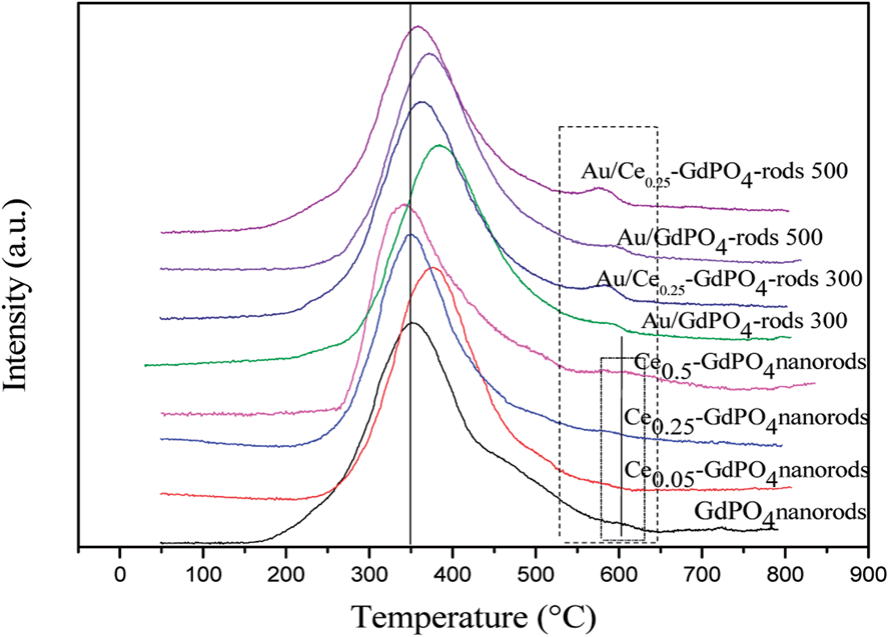
O_2_-TPD profiles of GdPO_4_ and Ce-GdPO_4_ composites and Au supported catalysts.

Fig. 8 shows the O_2_-TPD profiles of GdPO_4_ nanorods and Ce-GdPO_4_ composites, and the supported gold catalysts calcined at 300 °C. It can be seen that all the samples showed TPD peaks at 200–500 °C. After Au was deposited, a new desorption peak appeared at about 600 °C, indicating new active sites created in the Au supported catalysts. For pure GdPO_4_, a sharp TPD peak at ∼350 °C was detected. It could be found that after CePO_4_ was added, the desorption temperature of O_2_ shifted to high temperature for Ce_0.05_-GdPO_4_ nanorods. With increasing Ce contents, another weak desorption peak gradually appeared at about 600 °C attributable to representative of the desorption of strongly absorbed surface oxygen. It could be deduced that the modification of CePO_4_ to GdPO_4_ provided new O_2_ adsorption sites for the adsorption and activation of oxygen. The larger desorption peak, the stronger adsorption capacity of the sample to O_2_ and the more active sites. In CO catalytic oxidation process, CO molecules are usually adsorbed on the surface of Au. O_2_ is adsorbed on the surface of support or Au-support interface. The O_2_ desorption area increased in the order of Ce_0.50_-GdPO_4_ > Ce_0.25_-GdPO_4_ ≈ Ce_0.05_-GdPO_4_ > GdPO_4_, which indicated that the amount of surface-active oxygen species in Ce-GdPO_4_ is larger than that of the same species in GdPO_4_.^6,27*b*,30,31^ This might be related with the interaction between CePO_4_ and GdPO_4_. It might suggest that the addition of CePO_4_ has a significantly influence on the formation of O^2−^ species on the surface of support. Ref. 27*a* and *b* also pointed out that CePO_4_ could supply active oxygen vacancies which may promote CO oxidation. This was consistent with TPD results of Au/Ce-GdPO_4_-rods in this work, which means that the addition of CePO_4_ could promote the generation of active oxygen. CePO_4_ could promote the generation of active oxygen, confirming the synergistic interaction in the Ce-GdPO_4_ composite. After the deposition of Au, the position of the TPD peaks for 0.5% Au/Ce_0.25_-GdPO_4_-rods and 0.5% Au/GdPO_4_-rods calcined at 300 °C shifted to higher temperature than their support. It revealed that the deposition of Au gives rise to O_2_-adsorption sites at metal-support interface for the adsorption and activation of O_2_ molecules. This result could be also confirmed with the TPD data of the catalysts calcined at 500 °C. Due to the synergistic effect in Au/CePO_4_-GdPO_4_-rods, fluidity of active O was enhanced, leading to the shift of desorption temperature, which is beneficial to CO oxidation. The results showed that CePO_4_ can interact with GdPO_4_ promoting the formation of active sites. The deposition of Au made the desorption of O_2_ more difficult. It means that the adsorption capacity of the catalyst for O_2_ is increased, and the activity of the catalyst may be changed.

### CO_2_-TPD

**Fig. 9:**
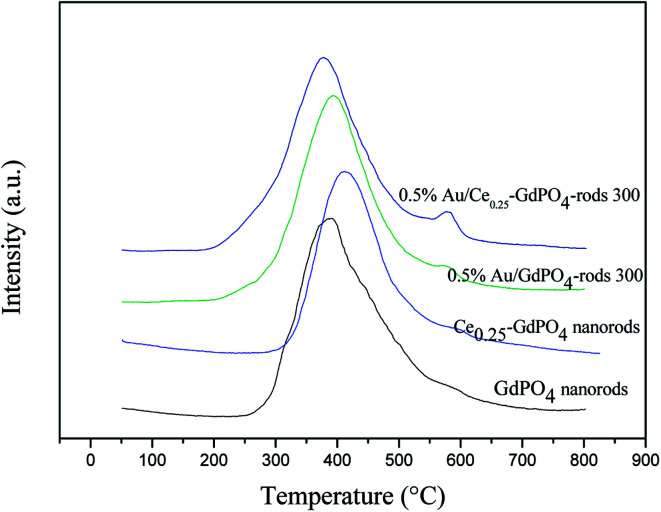
CO_2_-TPD Profiles of the supports and Au supported catalysts.

Similar conclusion was obtained with CO_2_-TPD Profiles of samples in Fig. 9 and S4.† In Fig. 9, the CO_2_ desorption temperature followed the order of Ce_0.25_-GdPO_4_ > 0.5% Au/GdPO_4_-rods > GdPO_4_ > 0.5% Au/Ce_0.25_-GdPO_4_-rods. Comparing with 0.5% Au/Ce_0.25_-GdPO_4_-rods, 0.5% Au/GdPO_4_-rods catalysts showed higher desorption temperature at ∼420 °C. This was an important indication of CO_2_ desorption ability of the samples, showing the major modifications of CePO_4_ and Au for their interaction. The strong adsorption of CO_2_ usually lowers the activity in catalysis.^27*a*^ As shown in Fig. S4,† it could be found that there was few CO_2_ absorbed on Au/CePO_4_ relatively, which might facilitate the good stability of the catalysts. The CO_2_ adsorption capacity of these catalysts was different from each other indeed. It would be synergistic interaction of CePO_4_ led to fewer basic sites and lower basicity of 0.5% Au/Ce_0.25_-GdPO_4_-rods compared to 0.5% Au/GdPO_4_-rods. From the results we can conclude that there were more basic sites in 0.5% Au/GdPO_4_-rods than in 0.5% Au/Ce_0.25_-GdPO_4_-rods. CO_2_ is more easily absorbed on 0.5%Au/GdPO_4_-rods than 0.5% Au/Ce_0.25_-GdPO_4_-rods. Thus, CO_2_ would desorb from active sites more difficultly in 0.5% Au/GdPO_4_-rods than in 0.5%Au/Ce-GdPO_4_-rods leading to the easy production of carbonate species, which might result in the deactivation of the catalysts.^27*a*^

**Fig. 10:**
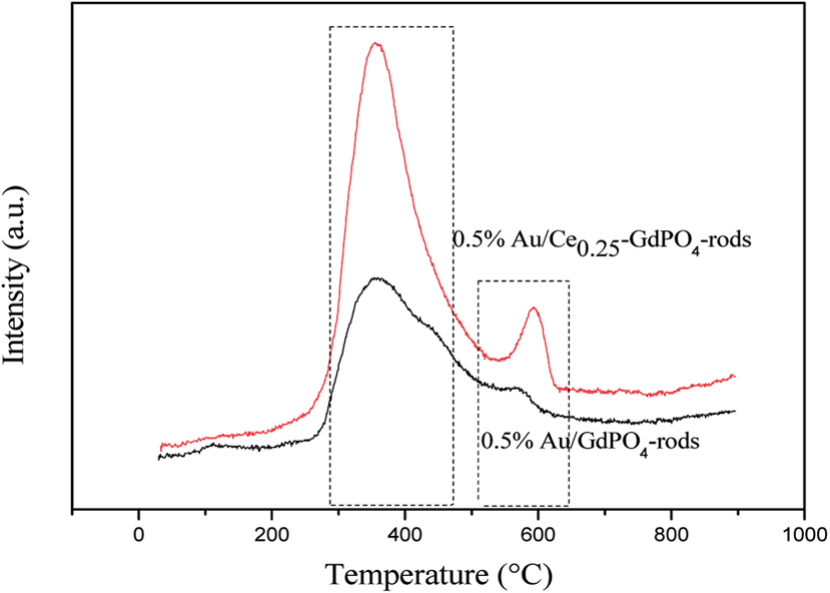
CO-TPD profiles of the 0.5% Au/GdPO_4_-rods and 0.5% Au/Ce_0.25_-GdPO_4_-rods catalysts.

### CO-TPD

CO-TPD analysis was also chosen to investigate the ability of CO adsorption on the catalysts. The CO-TPD spectra of Au/GdPO_4_-rods and Au/Ce-GdPO_4_-rods are shown in Fig. 10. The samples both displayed clear peaks at ∼400 °C, and a small peak in the range of 550–650 °C. But the desorption peaks for Au/Ce-GdPO_4_-rods are sharper than Au/GdPO_4_-rods indicating the stronger CO adsorption ability than Au/GdPO_4_-rods. Furthermore, in the range of 550–650 °C, the desorption temperature and the peak area of Au/Ce-GdPO_4_-rods were much higher and larger than that of Au/GdPO_4_, also indicative of the stronger CO adsorption on the Au/Ce-GdPO_4_-rods catalyst. In general, the stronger the adsorption of CO, the more number of active sites the catalysts owned.^7,13^ It's clear Au/Ce-GdPO_4_-rods had more active sites than Au/GdPO_4_-rods. The above characterization results showed that synergistic effect might exist in Ce-GdPO_4_-rods.

### XPS

**Fig. 11:**
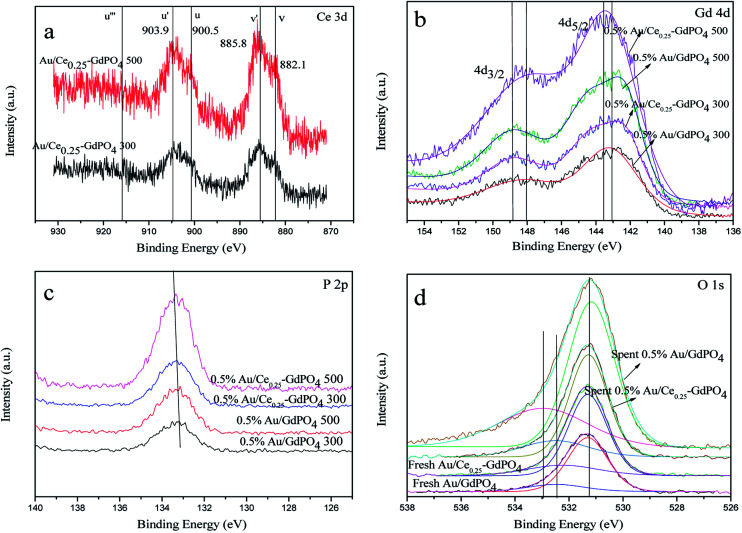
XPS spectra over fresh and spent 0.5% Au/GdPO_4_-rods and 0.5% Au/Ce_0.25_-GdPO_4_-rods calcined at 300 °C or 500 °C for 2 h of Ce 3d (a), Gd 4d (b), P 2p (c), O 1s (d).

XPS patterns were obtained to study the surface electronic states of Gd, Ce, P, O, and Au. As expected, the curve fitting analysis indicated that Ce (+3), Gd (+3), P (+5) and O (−2) species were simultaneously present in the samples. As shown in Fig. 11a, the XPS spectra of Ce 3d showed the distinct peaks of 3d_3/2_ spin–orbit states and 3d_5/2_ spin–orbit states. As defined in the picture, *v*, *v*′ centered at 882.1 and 885.8 eV represent the Ce 3d_5/2_ contributions, and *u*, *u*′ centered at 900.5 and 903.9 eV represent 3d_3/2_ contributions, which are the characteristic peaks for trivalent states of Ce^3+^. There was no distinct peaks detected at about 916 eV (*u*′′′, being characteristic of Ce^4+^), which arises from a transition of the 4 f′′ final state from the 4 f′′ initial state, suggesting no existence of Ce^4+^.^3,7,15,17,29^ Since it is absent in Au/Ce-GdPO_4_, combining with The signals *v*′ and *u*′, it could be deduced that Ce is mainly present as Ce^3+^ in the samples. In Fig. 11b, for 0.5% Au/GdPO_4_-rods, Gd 4d peaks centered at 143.1 (Gd 4d_5/2_) and 148.2 eV (Gd 4d_3/2_) indicating that Gd ions are Gd^3+^.^3,41,42^ However, the values of binding energy of Gd 3d_3/2_ for 0.5% Au/Ce_0.25_-GdPO_4_-rods calcined at 300 and 500 °C were about 0.4 eV lower than that for Au/GdPO_4_, which could be assigned to Gd ions embedded in CePO_4_ nanorods due to the interaction of CePO_4_ with GdPO_4_. As shown in Fig. 11c, the position of P 2p peak at about 133.4 eV indicated the presence of P(v) species which was the evidence for the formation of P–O chemical bonding in this report.^33,39,40^ The relatively lower binding energy of the P 2p for Au/Ce-GdPO_4_ calcined at 300 °C and 500 °C than that for Au/GdPO_4_ may also suggest that the Gd ions interact with CePO_4_. It is obviously concluded that CePO_4_ and GdPO_4_ were incorporated together due to the interaction of CePO_4_ with GdPO_4_.^33^ For the fresh 0.5% Au/GdPO_4_-rods and 0.5% Au/Ce_0.25_-GdPO_4_-rods, a broad O 1s XPS peak (Fig. 11d) was found at 530 eV, attributed O of PO_4_^3−^.^33,36–40^ The peak at around 532 eV could be assigned to surface absorbed O^*δ*−^ species such as OH^−^.^3^ But for the spent catalysts, the peak shifted to about 533 eV in the O 1s XPS profile of spent 0.5% Au/GdPO_4_-rods, which was not observed for spent 0.5% Au/Ce_0.25_-GdPO_4_-rods. This peak could be attributed to the oxygen in the carbonate species present on the surface of the surface. Though it couldn't be the evidence to demonstrate the formation of the carbonate species in the reaction, there had been new products which might be bad for enhancing the activity of the catalysts generated.

### FT-IR

**Fig. 12:**
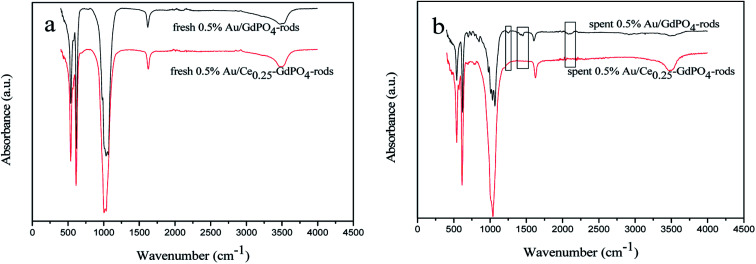
FT-IR spectra of 0.5% Au/GdPO_4_-rods (a) and 0.5% Au/Ce_0.25_-GdPO_4_-rods (b) calcined at 300 °C for 2 h.

**Fig. 13:**
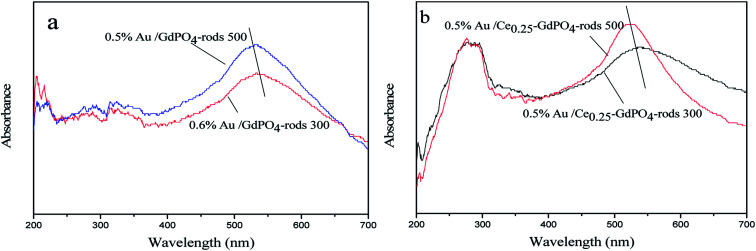
UV-Vis spectra of 0.5% Au/GdPO_4_-rods (a) and 0.5% Au/Ce-GdPO_4_-rods (b) calcined at 300 and 500 °C for 2 h.

FT-IR spectra of the fresh and spent catalysts were presented in Fig. 12 to give insight into interaction between CePO_4_ and GdPO_4_. For the 0.5% Au/Ce_0.25_-GdPO_4_-rods catalysts, as shown in Fig. 13a, the distinctive absorption peaks at about 542, 611, 1040, 1617, and 3506 cm^−1^ were ascribe to asymmetric stretching and bend vibrations of PO_4_^3−^.^34,38,43^ For the spent catalysts, there was no new absorption peaks appeared compared with the fresh 0.5% Au/Ce_0.25_-GdPO_4_-rods. However, in Fig. 13b, the obvious IR adsorption peaks at 1256, 1470 and 2066, 2100 cm^−1^ for spent 0.5% Au/GdPO_4_-rods were indexed as the *υ*(CO_3_) of bicarbonate or bidentate carbonate species and absorbed CO compared with fresh 0.5% Au/GdPO_4_-rods.^1,5,27*b*,34^ It revealed that there were carbonate species formed on the surface of Au/GdPO_4_ after combustion for CO oxidation. It has been reported that the accumulation of carbonate species could block the active sites leading to the deactivation of catalyst resulting in the deactivation of 0.5% Au/GdPO_4_-rods. As expected, 0.5% Au/GdPO_4_-rods showed lower stability than 0.5% Au/Ce_0.25_-GdPO_4_-rods in the reaction of CO oxidation.

### UV-Vis

The small size and high dispersion of Au nanoparticles in Au/GdPO_4_-rods and Au/Ce-GdPO_4_-rods were also confirmed by the UV-Vis spectra (Fig. 13). As seen in the figure, compared with the support, the catalysts shown obvious band centered at approximately 500–600 nm, which is the plasmon resonance of Au particles.^26,35^ As observed, the catalysts calcined at 300 °C showed the broader and red-shifted plasmon resonance band compared with the catalysts calcined at 500 °C. The intensity of the band also performed lower than that of 500 °C pretreated catalysts. The size, content and shape of Au particles and interaction with the support, which would change the electronic properties of the surrounding environment, usually bear the primary responsibility for the red shift.^26,35^ While the size of Au nanoparticles changed, a reduction of electron density in Au particles occurred, leading to the transition of electrons from Au cluster to the surrounding matrix, changing the interaction between Au and support, leading to the shift of SPR band. This was well in line with results of our catalysts calcined at different temperatures. The red shift observed in the present case might be due to this transferred-electron effect. It's known that the high temperature calcination would facilitate the produce of strong metal-support interaction. Combined with TEM characterization results described above, it could be suggested gold particles in the catalysts calcined at 300 °C had small size, and Au-support interaction was enhanced with increasing the calcination temperature.

### CO oxidation

**Fig. 14:**
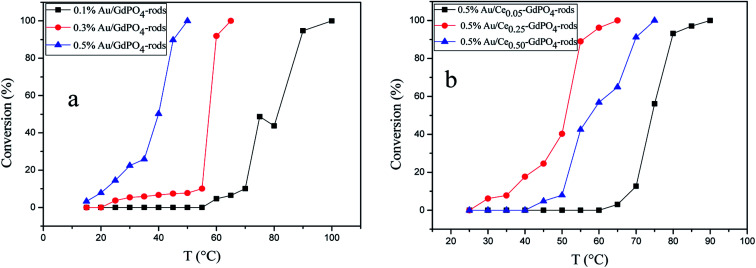
CO conversion over Au/GdPO_4_-rods calcined 300 °C for 2 h with different gold loadings: 0.1%, 0.3%, 0.5% (a), and 0.5% Au/Ce-GdPO_4_-rods with the Ce contents of 5 at%, 25 at%, 50 at% (b).

The CO conversion as a function of temperature catalyzed by the Au/GdPO_4_-rods and 0.5% Au/Ce-GdPO_4_-rods is shown in Fig. 14. The CO conversion results for Au/GdPO_4_-rods pretreated at 300 °C are shown in Fig. 14a. The total conversion temperatures of Au/GdPO_4_-rods catalysts (gold content: 0.1, 0.3, 0.5%) were 90, 65, 50 °C, respectively. It was noteworthy that the Au/GdPO_4_-rods catalysts showed good activity for CO oxidation with low content of gold. After adding CePO_4_ to GdPO_4_ (Fig. 14b), the catalytic activity decreased greatly. The total CO conversion temperature increased in the order of 0.5% Au/Ce_0.50_-GdPO_4_-rods > 0.5% Au/Ce_0.05_-GdPO_4_-rods > 0.5% Au/Ce_0.25_-GdPO_4_-rods. While the amount of Ce was 25%, the 0.5% Au/Ce_0.25_-GdPO_4_-rods showed the best activity with the complete CO conversion temperature of 65 °C, which was similar to that of 0.5% Au/GdPO_4_-rods. In comparison, the *T*_100%_ of the catalysts increased as the order: 0.5% Au/GdPO_4_-rods > 0.5% Au/Ce-GdPO_4_-rods > 0.5% Au/CePO_4_-rods.^37^ The results suggested that the activity of Au/Ce-GdPO_4_-rods might be mainly from active Au species on Ce-GdPO_4_ and CePO_4_ nanorods. The addition of CePO_4_ promoted the produce of strong interaction in the catalysts. The TOFs of 0.5% Au/GdPO_4_-rods and 0.5% Au/Ce_0.25_-GdPO_4_-rods were 1.94 s^−1^ and 1.64 s^−1^ at the reaction temperature of 55 °C (Table S1†). The catalytic activity of 0.5% Au/Ce_0.25_-GdPO_4_-rods and 0.5% Au/GdPO_4_-rods is also compared with other catalysts reported in literatures. 0.5% Au/Ce_0.25_-GdPO_4_-rods catalyst showed higher TOF than Au/CePO_4_-rods and Au/LaFeO_3_-MCF-0.6.^18^ The high activity of Au depends strongly on the addition of CePO_4_, owning to the synergistic interaction between Au and supports. This indicates that Ce^3+^ concentration plays a very important role for determining the catalytic activity. As known, the nature of the support could significantly affect the activity of gold catalysts.^11,22,27*b*^ After the addition of Ce, the BET surface area of GdPO_4_ slightly increased. The TPD results revealed that due to the synergistic interaction in the Ce-GdPO_4_ composite, 0.5% Au/GdPO_4_-rods could adsorb more O_2_ than 0.5% Au/Ce_0.25_-GdPO_4_-rods. But 0.5% Au/Ce_0.25_-GdPO_4_-rods could adsorb more CO and less CO_2_ than 0.5% Au/GdPO_4_-rods. The particle diameter was another vital factor determining the activity of gold catalysts. The TEM data showed that Au mean size of 0.5% Au/GdPO_4_-rods was smaller than that of 0.5% Au/Ce_0.25_-GdPO_4_-rods calcined at 300 °C, while the Au particles with the size of <5 nm would perform better catalytic activity than bigger ones. Due to the difference of the supports, 0.5% Au/GdPO_4_-rods could provide more active sites than 0.5% Au/Ce_0.25_-GdPO_4_-rods. Thus, the catalyst could have better activity. The appropriate amount of Ce was 25%. Au loading efficiency of Au/Ce-GdPO_4_-rods was also higher than Au/GdPO_4_-rods. TEM data (shown in Fig. S2†) revealed that CePO_4_ could disperse well in GdPO_4_ and strongly interact with GdPO_4_. Then combined with O_2_-TPD results, it could be concluded that strong synergistic effect existed in the catalysts which could help immobilize Au particles on the interface of CePO_4_–GdPO_4_ favoring the reduction of the support, thus allowing lattice oxygen atoms from the support to become activated species available for CO oxidation. As known, Au particle size was another vital factor in determining the activity of Au supported catalysts. Here, the mean size of Au particles in Au/Ce-GdPO_4_-rods was slightly larger than that in Au/GdPO_4_-rods. In union of the two decisive effects, Au/Ce-GdPO_4_-rods showed relatively weaker activity than Au/GdPO_4_-rods.

**Fig. 15:**
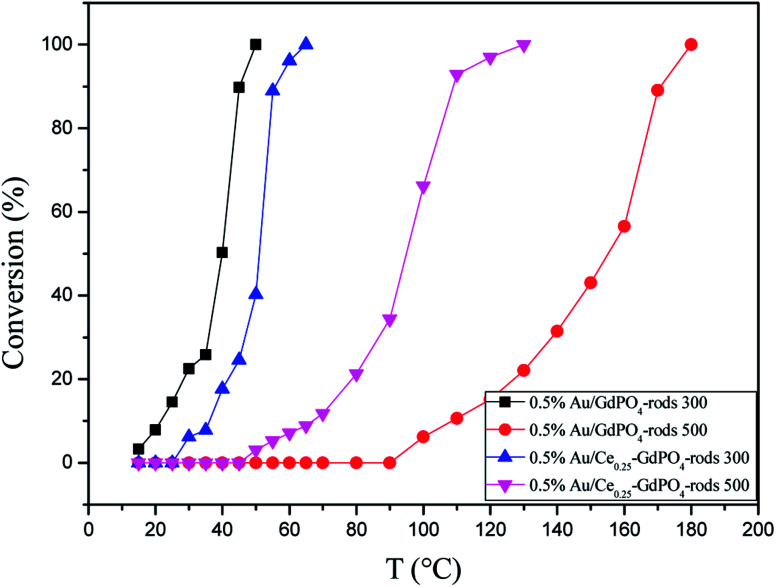
CO conversion over 0.5% Au/GdPO_4_-rods and 0.5% Au/Ce_0.25_-GdPO_4_-rods calcined 300 °C or 500 °C for 2 h.

Fig. 15 compared the activity of 0.5% Au/GdPO_4_-rods and 0.5% Au/Ce_0.25_-GdPO_4_-rods after calcination at 500 °C for 2 h. It's clear that the activity of both catalysts decreased. However, it decreased severely for 0.5% Au/GdPO_4_-rods with values of TOF (0.28 s^−1^) much smaller than 0.5% Au/Ce_0.25_-GdPO_4_-rods (1.77 s^−1^). It could be seen form the results of TEM that the diameter of Au particles in 0.5% Au/GdPO_4_-rods pretreated at 500 °C was about 10 nm which was much bigger than 7 nm for 0.5% Au/Ce_0.25_-GdPO_4_-rods. As known, the particle diameter of Au particles was a vital factor in determining the activity of gold catalysts. The BET results also indicated that after high temperature treatment, the surface area of 0.5% Au/GdPO_4_-rods decreased more than that of 0.5% Au/Ce_0.25_-GdPO_4_-rods. The TPD results also showed that 0.5% Au/Ce_0.25_-GdPO_4_-rods calcined at 500 °C could supply more active sites than 0.5% Au/GdPO_4_-rods. It was clear that these characteristics on Au/Ce-GdPO_4_-rods were different from those on Au/GdPO_4_-rods, which showed weak high-temperature resistance. These results might explain that 0.5% Au/Ce_0.25_-GdPO_4_-rods calcined at 500 °C possessed better activity than 0.5% Au/GdPO_4_-rods. The relative high catalytic performance 0.5% Au/Ce_0.25_-GdPO_4_-rods calcined at 500 °C were also comparing the activities calculated at 80 °C in the literatures which was similar to Au/TiO_2_ (2.8 s^−1^) and Au/Ce–K-OMS-2 (2.3 s^−1^).^14,15^ Indeed, it was the interaction between CePO_4_ and GdPO_4_ which fabricate the supported Au nanoparticles improving sintering resistance. And the Au active species on the interface of CePO_4_–GdPO_4_ made important contribution to the high-temperature resistance of Au/Ce-GdPO_4_-rods. So it could be concluded that the synergistic interaction of CePO_4_ could help stabilize Au nanoparticles and prevent the agglomeration of Au nanoparticles.

**Fig. 16:**
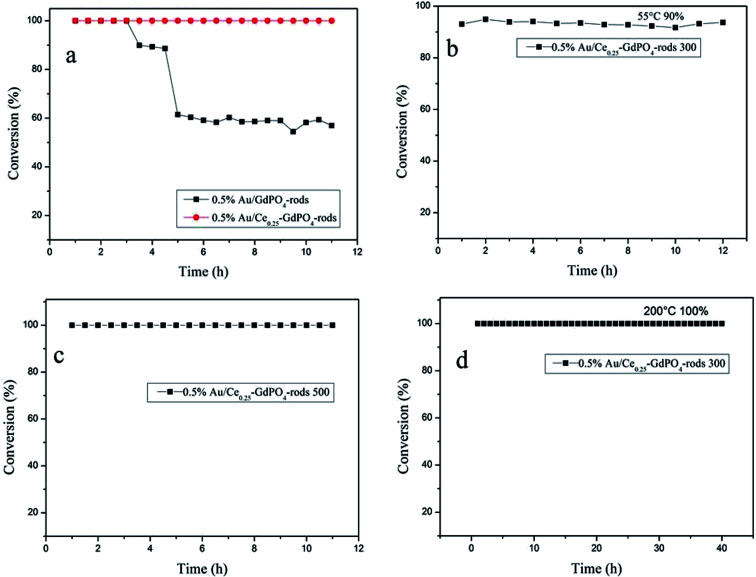
The stability for the CO oxidation of 0.5% Au/GdPO_4_-rods, 0.5% Au/Ce_0.25_-GdPO_4_-rods (*T*_100%_ = 50 °C and 65 °C, respectively) (a), 0.5% Au/Ce_0.25_-GdPO_4_-rods, *T*_90%_ = 55 °C calcined at 300 °C for 2 h (b), 0.5% Au/Ce_0.25_-GdPO_4_-rods calcined at 500 °C, *T*_100%_ = 130 °C (c), 0.5% Au/Ce_0.25_-GdPO_4_-rods calcined at 300 °C, reaction temperature: 200 °C (d).

As known, not only the activity of supported gold catalysts is of great importance but also the stability.^2,22,26,27*b*^ The stability tests were also performed for 0.5% Au/GdPO_4_-rods and 0.5% Au/Ce_0.25_-GdPO_4_-rods in comparison to study the synergistic effect, maintaining a continuous CO oxidation reaction at temperature of 100% CO conversion for 12 h respectively. Fig. 16a shows the deactivation curves of the two catalysts. The data revealed that 0.5% Au/GdPO_4_-rods showed apparent deactivation in 12 h. The CO conversion decreased with 0.5% Au/GdPO_4_-rods from 100% to 90% in the first 3 h, and after another 3 h, CO conversion was kept on decreasing to 40% deactivation. But the deactivation for 0.5% Au/Ce_0.25_-GdPO_4_-rods was different. It could be obviously found that in the 12 h of reaction time, CO conversion did not decrease. At the reaction temperature of 55 °C (Fig. 16b), after continuous reaction for 12 h, CO conversion of 0.5% Au/Ce_0.25_-GdPO_4_-rods decreased only 2% (from 90% to 88%) of its initial activity. After the calcinations at 500 °C (Fig. 16c), the 0.5% Au/Ce_0.25_-GdPO_4_-rods could also exhibit good stability with the conversion of 100% at 130 °C for 12 h without any deactivation. Combined with the TEM data, FTIR data and the activity results of the catalysts calcined at 500 °C, the deactivation of 0.5% Au/GdPO_4_-rods might be attributed to the sintering of the gold nanoparticles and adsorption of carbonates on the active sites. It is noted that there were more carbonates on the surface of spent 0.5% Au/GdPO_4_-rods than on the spent 0.5% Au/Ce_0.25_-GdPO_4_-rods catalysts. Au nanoparticles in spent 0.5% Au/GdPO_4_-rods aggregated more severely than in spent 0.5% Au/Ce_0.25_-GdPO_4_-rods. The carbonates might block the active sites resulting in the deactivation of the catalysts.^27*b*,35^ We also tested the stability of Au/Ce_0.25_-GdPO_4_-rods at high temperature for 50 h. As shown in Fig. 16d, the catalyst did not lose its activity with CO conversion of 100% during the continuous reaction even at temperature of 200 °C. It's clear that 0.5% Au/Ce_0.25_-GdPO_4_-rods with the better stability and better high temperature resistance has smaller Au particles and fewer carbonates. The results presented a similar conclusion that the synergistic interaction between CePO_4_ and GdPO_4_ could help stabilize Au particles and account for the good stability of 0.5% Au/Ce_0.25_-GdPO_4_-rods. Compared with Au/Ce-GdPO_4_-rods, Au/GdPO_4_-rods showed better activity but lower stability and weaker high-temperature resistance. Combined with characterization data, it should have important relation with synergistic effect in Au/Ce-GdPO_4_-rods. For instance, stability tests of Au/Ce-GdPO_4_-rods at other low temperatures might be also performed to justify the activity enhancement of CePO_4_ additive. The synergistic effect between Au and supports would be also studied deeply in the further research supporting by some solid characterization data (*e.g.* in suit FT-IR).

As discussed above, we could finally draw the conclusion that there may be three sorts of active component (1) Au supported on CePO_4_, owning high loading efficiency and small particle size; (2) Au deposited on CePO_4_–GdPO_4_ interface, which possessing good activity and stability due to the strong synergistic effect between Au and Ce-GdPO_4_ or CePO_4_ in the catalysts; (3) Au on GdPO_4_, suffering from deactivation owning to the block of the active sites by carbonates resulting from the reaction. The first two functions made relative more contribution to catalytic CO oxidation than the third one depending on the promotional effect of CePO_4_.

## Conclusion

In summary, a new Ce-GdPO_4_ composite was developed. The strong interaction of CePO_4_ in the composite could account for the sintering resistance and stability of the Au nanoparticles. The CO catalytic tests showed that the Au/GdPO_4_-rods catalyst with low Au content still has good catalytic performance. It's obvious that after high temperature calcination, Au/Ce-GdPO_4_-rods showed better activity than Au/GdPO_4_-rods. And Au/Ce-GdPO_4_-rods also possessed good durability. CePO_4_ could effectively improve sintering resistance and stability of Au nanoparticles in the catalyst because CePO_4_ as auxiliary for GdPO_4_ could make Au fixed on the surface of the support, and inhibited the aggregation of Au nanoparticles. In all, Au/GdPO_4_-rods could be good catalysts with low content of Au, but the catalyst showed bad sintering resistance to high temperature and poor durability. However, the designed Au/Ce-GdPO_4_-rods catalyst by a grinding-ultrasonic method possessed good catalytic activity, good sintering-resistance and excellent stability. The enhancement of activity by the dopant of CePO_4_ is related to synergistic effect between CePO_4_ and GdPO_4_ or Au and the support. This physical mixing method is an effective procedure. In addition, it significantly has the value of practical applications.

## Conflicts of interest

There are no conflicts to declare.

## Supplementary Material

RA-008-C8RA02206B-s001
