# Supplementary material for: The influence of CePO4 nanorods on the CO oxidation activity of Au/GdPO4-rods
Source: RSC Adv. 2018 Jun 13;8(39):21699–711. doi: 10.1039/c8ra02206b (PMC9080986; doi:10.1039/c8ra02206b)
Supplement: RA-008-C8RA02206B-s001 [file RA-008-C8RA02206B-s001.pdf]

Electronic Supplementary Material (ESI) for RSC Advances.
This journal is © The Royal Society of Chemistry 2018

Supporting Information for

The influence of $CePO_4$ nanorods on the CO oxidation activity of Au/$GdPO_4$-rods

Yu Huanhuan[a, *], Chen Fayun[a], Zhubaolin[b], Huang Weiping[b], and Zhang Shoumin[b,§]

[a] College of Chemistry and Environmental Science, Shangrao Normal University, Shangrao 334001, P. R. China

[b] Department of Chemistry, Key Laboratory of Advanced Energy Material Chemistry (MOE), and TKL of Metal and Molecule Based Material Chemistry, Nankai University, Tianjin 300071, P. R. China

* Corresponding author. Tel: +8615620206206; E-mail: yuhuanhuan08@163.com

§ Corresponding author. Tel: +8613920779712; E-mail: zhangsm@nankai.edu.cn

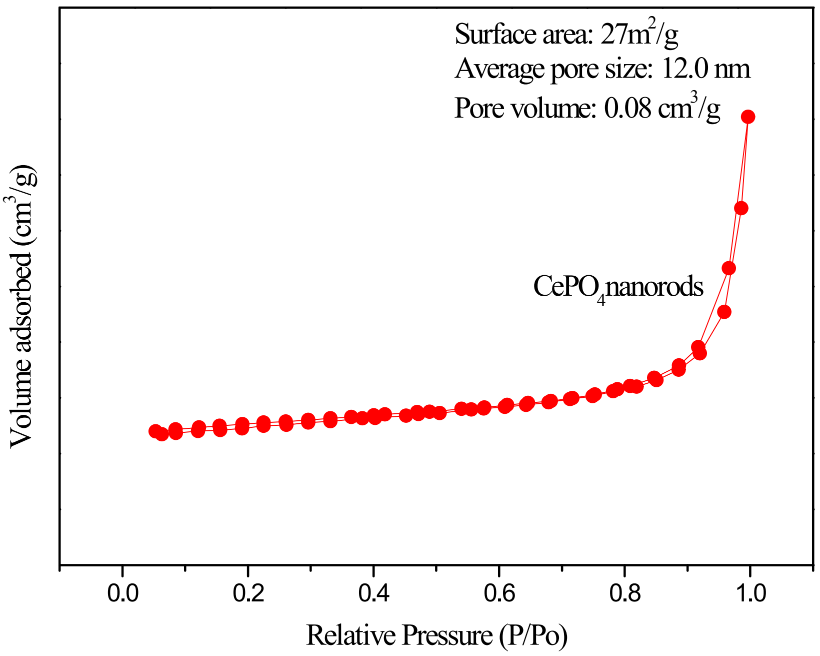

Fig. S1 BET results of $CePO_4$ nanorods [37]

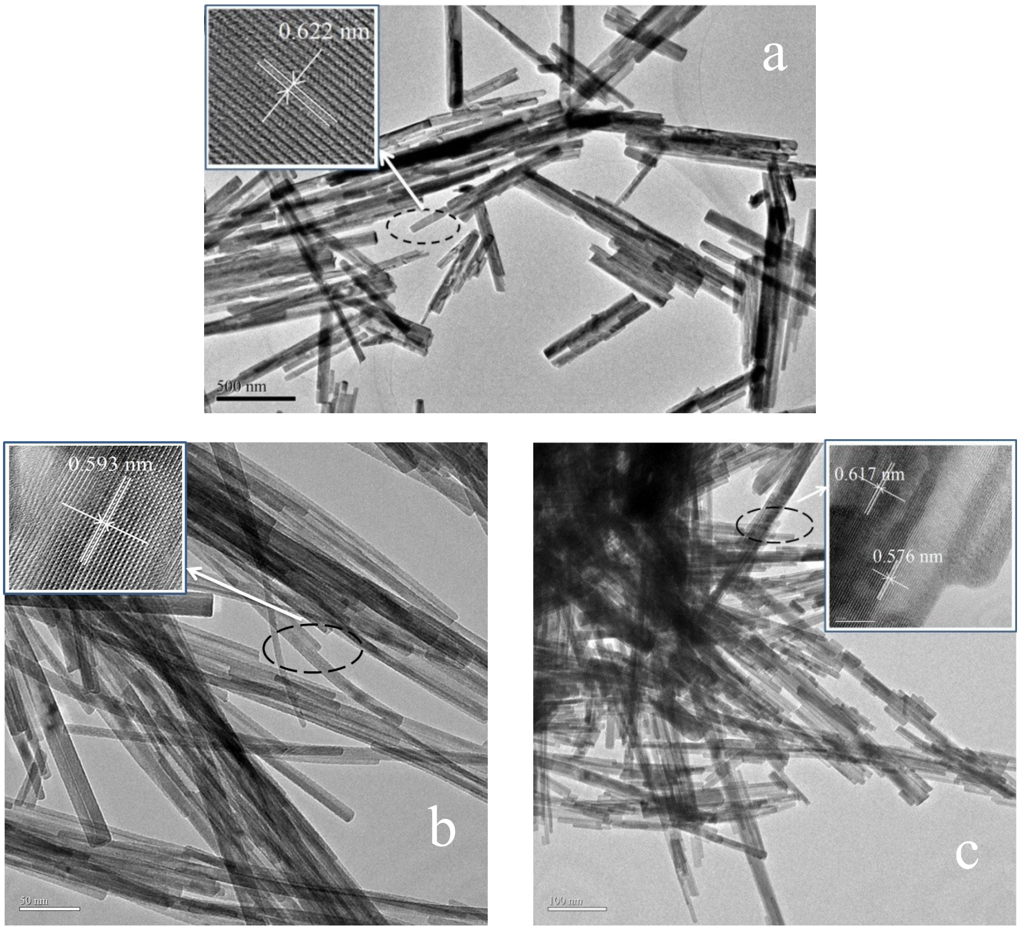

Fig. S2 TEM images of $CePO_4$-rods (a), $GdPO_4$-rods (b), and Ce-$GdPO_4$-rods (c)

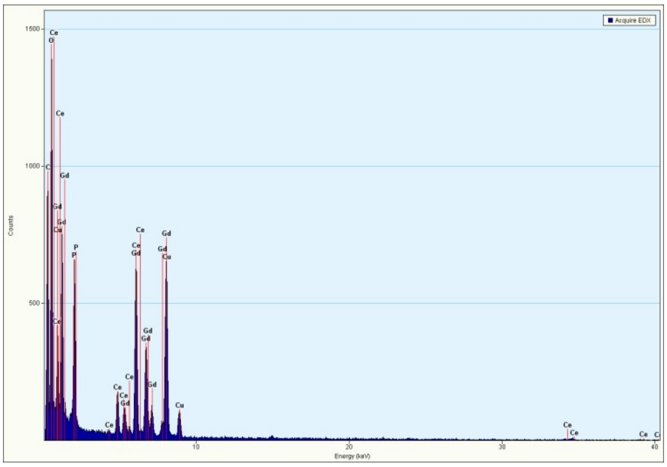

Fig. S3 EDX element analysis of Ce-$GdPO_4$ nanorods

As shown in Fig.S2, for $GdPO_4$ support, nearly uniform nanorod with a diameter of about 8-15 nm. The TEM image of $CePO_4$ showed the presence of $CePO_4$ nanorods with the diameter of about 15–20 nm. As seen from Fig. S2c, the $CePO_4$ nanorods were well-dispersed on the surface of $GdPO_4$ nanorods. Just from the morphology, it's really difficult to distinguish $CePO_4$ or $GdPO_4$. From the high-resolution (HR)-TEM images inset of Fig. S2a and b, the lattice fringes of 0.622 and 0.593 nm corresponded to the (100) planes of $CePO_4$ and (100) planes of $GdPO_4$, respectively.[39-40,S1-S2] In Fig. S2c, the HRTEM image taken from the interface of two single nanowires

showed the clearly resolved planes of (100). The lattice fringes of 0.617 and 0.576 nm corresponded to $CePO_4$ and $GdPO_4$, which were lower than pure $CePO_4$ and $GdPO_4$ respectively. Combined with the result of Fig. S3, it could be concluded that the $CePO_4$ nanorods highly dispersed among $GdPO_4$ nanorods. And interfaces between $CePO_4$ and $GdPO_4$ nanorods were involved in the Ce-$GdPO_4$ supports. Thus, after Au added, gold nanoparticles could not only deposite on the surfaces of $CePO_4$ and $GdPO_4$ nanorods but also on the interface of $CePO_4$-$GdPO_4$ enhancing the interaction between gold and supports.

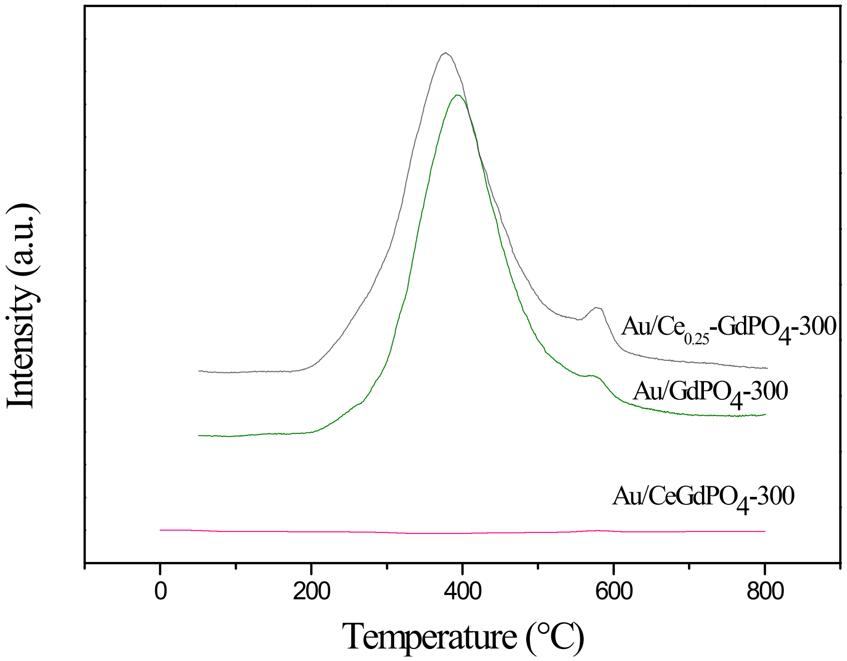

Fig. S4 CO_2-TPD profiles of 0.5% Au/$CePO_4$-rods, 0.5% Au/$GdPO_4$-rods and 0.5% Au/$Ce_{0.25}$-$GdPO_4$-rods calcined at 300 °C for 2 h

As seen in Figure S4, There was little CO_2 absorbed on Au/$CePO_4$, indicating that 0.5% Au/$CePO_4$-rods had more basic sites than 0.5% Au/$CePO_4$-rods and 0.5% Au/$Ce_{0.25}$-$GdPO_4$-rods. These basic sites would produced negative impact on responding catalytic activity for CO oxidation of the catalysts.

Table S1 Comparison of the activities of supported Au catalysts for CO oxidation with those reported in literatures

Catalyst	d (nm)	TOF (s^{-1})	Approximate Reaction rate (10^{-3} mol s^{-1} g^{-1}_{cat})	reaction remperature (°C)	Reference
0.5% Au/$GdPO_4$-rods-300	3.84	1.94	2.97	55	This work
0.5% Au/$Ce_{0.25}$-$GdPO_4$-rods-300	4.03	1.64	2.40	55	This work
0.5% Au/$GdPO_4$-rods-500	8.0	0.28	0.21	100	This work
0.5% Au/$Ce_{0.25}$-$GdPO_4$-rods-500	5.0	1.77	2.08	100	This work
0.5% Au/$CePO_4$-rods-300	4	0.21	0.31	55	[37]
Au/CeO_2	~5	/	10.7	55	[33]
Au/$LaFeO_3$-MCF-0.6	~5	/	0.43	50	[18]
Au/Ce–K-OMS-2	7.0	2.3	/	80	[15]
Au/TiO_2	2.5	2.8	5.1	80	[14]

Reference

[S1]W. Wang, X. P. Jiang and K. Z. Chen, *Chem. Commun.,* 2012, 48, 6839-6841

[S2]X. Y. Kuang, H. Liu, W. Y. Hua and Y. Z. Shao, *Dalton Trans.*, 2014, 43, 12321–12328.
